# Osteoid Osteoma of the Proximal Phalanx of the Great Toe in a 13-Year-Old Female Patient

**DOI:** 10.5435/JAAOSGlobal-D-21-00223

**Published:** 2023-03-06

**Authors:** Kyu Bum Seo, Seung Jin Yoo, Yong Yeon Chu, Chaemoon Lim

**Affiliations:** From the Department of Orthopedic Surgery, Jeju National University Hospital, Jeju, South Korea.

## Abstract

Osteoid osteoma (OO) is a benign osteoblastic bone tumor typically involving the diaphysis or metaphysis in long tubular bones. OO in phalanges of the great toe has been rarely reported, and it is often challenging to differentiate with subacute osteomyelitis, bone abscess, or osteoblastoma. This case report describes an uncommon case of a 13-year-old female patient with subperiosteal OO in the proximal phalanx of the great toe. The atypical location of OO should be familiarized to include appropriate differential diagnosis and to ensure accurate diagnosis by radiologic evaluations. Surgical excision remains the benchmark for the treatment of OO with its advantages on direct visualization and histologic confirmation for the diagnosis.

Osteoid osteoma (OO), a benign osteoblastic bone tumor typically involving the diaphysis or metaphysis of long tubular bones such as the femur or tibia, is responsible for approximately 10% of all benign bone tumors.^1^ OO mostly occurs in the second and third decades of life with male predilection. OO in phalanges of the great toe has been rarely reported in the previous literature to the best of our knowledge, and it is often challenging to differentiate with subacute osteomyelitis, bone abscess, or osteoblastoma.^2^ In this report, we present an uncommon case of a 13-year-old female patient with subperiosteal OO in the proximal phalanx of the great toe.

## Case Report

A 13-year-old female patient was admitted to the department of orthopaedic surgery outpatient clinic with a chief complaint of recurring swelling, burning sensation, and focal tenderness at the right great toe for the past 12 months with no history of trauma. Her symptoms had been well-controlled by NSAIDs but reappeared 4 months ago. The pain was more severe at night after long periods of walking or sports activities.

**Figure 1:**
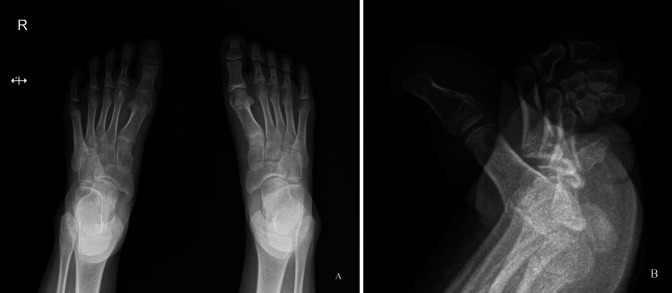
Simple anterioposterior (**A**) and lateral (**B**) radiographs of the right foot showing a 4-mm, round, radiolucent lesion with central mineralization and rim sclerosis at the head of the proximal phalanx in the great toe.

**Figure 2:**
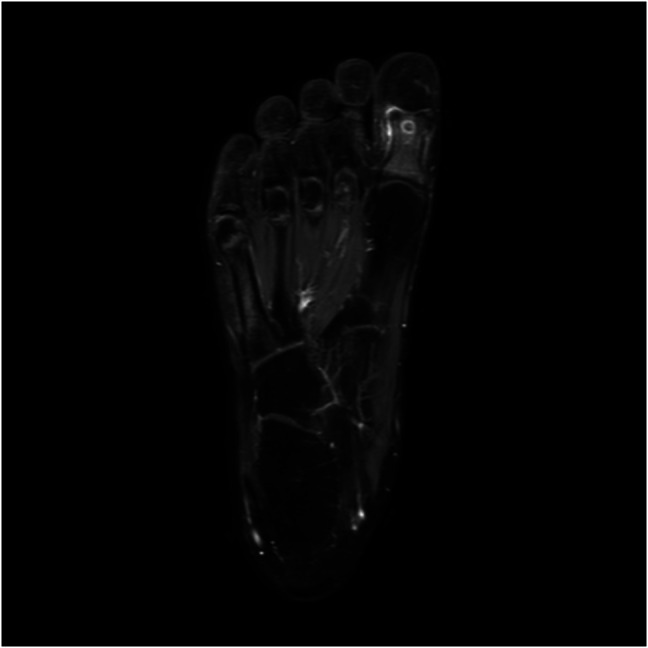
Gadolinium-enhanced MR T1-weighted axial images indicating a 4-mm bony lesion with high signal intensity in the periphery and low signal intensity at the center along with diffuse bone marrow edema and periosteal inflammatory changes in the proximal phalanx of the great toe.

**Figure 3:**
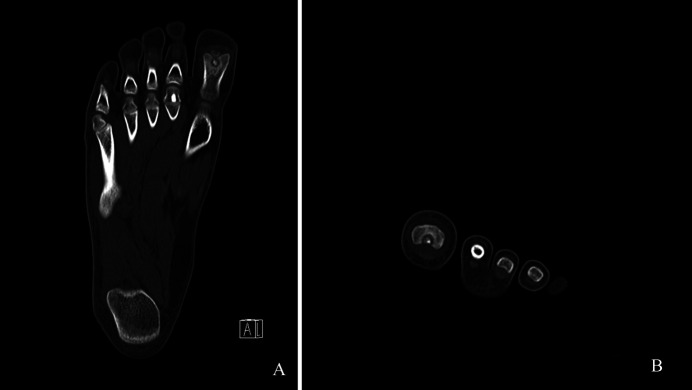
Computed tomography axial (**A**) and coronal (**B**) images showing a radiolucent central nidus surrounded by sclerotic rim in the head of the proximal phalanx of the great toe.

The physical examination revealed a slightly swollen great toe with focal tenderness at the interphalangeal joint area of the plantar aspect of the great toe. The radiographs showed a 4-mm, round, radiolucent lesion with central mineralization and rim sclerosis at the head of the proximal phalanx of the great toe (Figure 1). The complete blood analysis indicated no remarkable findings. With a clinical suspicion of subacute osteomyelitis, enhanced MRI indicated heterogeneously low signal intensity on T1-weighted images and low signal intensity in the center and higher signal intensity in the periphery on T2-weighted images at the same lesion seen on the simple radiograph. In addition, there were diffuse bone marrow edema and periosteal inflammatory change around the bony lesion along with thin enhancement at the periphery of the lesion (Figure 2). CT revealed a radiolucent central nidus surrounded by a sclerotic rim (Figure 3). Both MRI and CT findings were consistent with the diagnosis of OO in the head of the proximal phalanx of the great toe. After nonsurgical management with pain medication for 2 months, the patient underwent surgical excision because of recurring pain.

**Figure 4:**
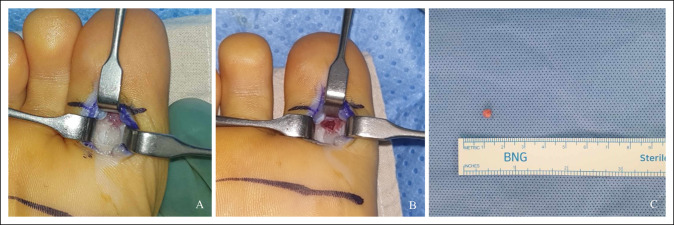
Intraoperative clinical photographs showing a grayish red and gritty lesion in the head of the proximal phalanx of the great toe (**A**). Postexcision intraoperative clinical photographs indicating a complete removal of the bone tumor after surgical excision and curettage (**B**) and the resected bone tumor specimen (**C**).

**Figure 5:**
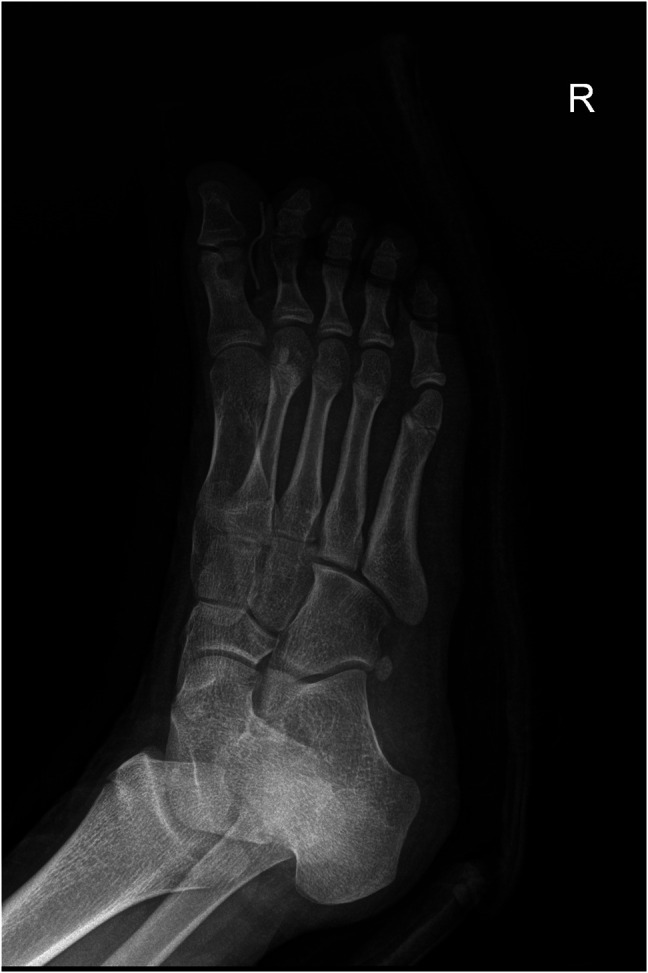
Immediate postoperative radiograph indicating complete resection of the bone tumor in the proximal phalanx of the great toe.

Under general anesthesia, the patient was positioned prone for better access to the plantar location of OO. A longitudinal incision was made on the plantar aspect of the great toe, and after retracting the flexor hallucis longus tendon laterally, the subperiosteally located bony lesion was visible. Intraoperative localization of the nidus was rather direct and simple because of its juxtacortical and subperiosteal location without additional damages to the surrounding osseous structure. A 5-millimeter nidus was completely removed with en bloc surgical resection with curettage and sent for microbiologic and histopathologic analyses (Figure 4). The immediate postoperative radiograph showed complete excision of the nidus in the proximal phalanx of the great toe (Figure 5).

**Figure 6:**
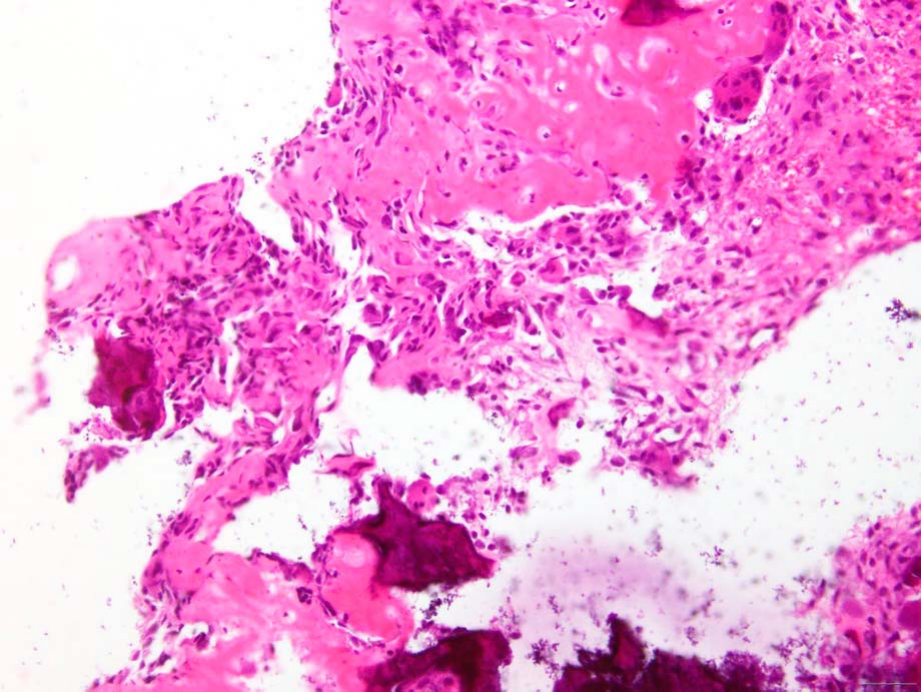
An osteoid osteoma nidus and the normal bone structures are shown in the permanent biopsy of the bone tumor. The hematoxylin and eosin–stained section showing the hypercellular nidus with vascular fibrous tissues and prominent osteoblastic rims (magnification, ×400)

**Figure 7:**
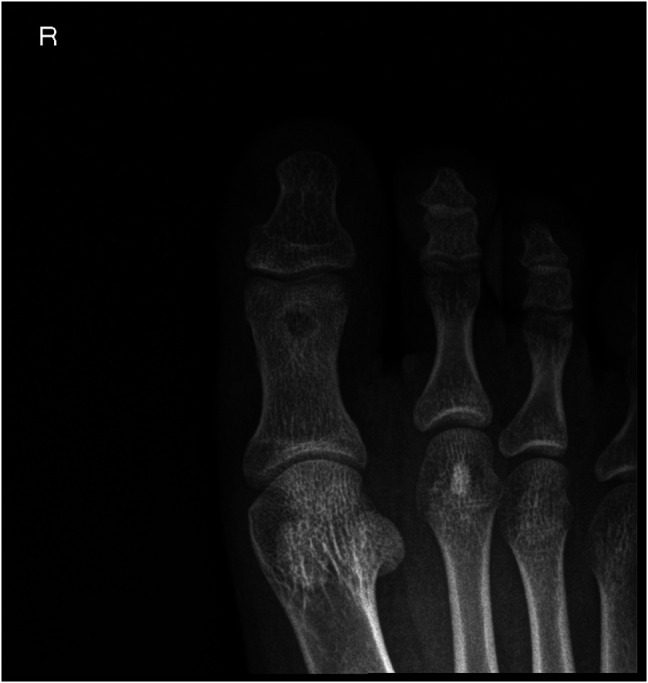
Follow-up radiograph obtained at 1 year after the surgical excision revealed no signs of recurrence in the proximal phalanx of the great toe.

Histopathologic examination revealed OO with typical morphologic features of hypercellular central nidus with vascular fibrous tissues, surrounded by osteoblastic rims (Figure 6). Microbiologic culture showed no pathogenic organisms present. The patient's symptoms remitted immediately after the surgery, and the patient postoperatively began weight-bearing with postoperative flat-soled shoes for 2 weeks. One year after the surgical resection, the follow-up radiograph indicated no recurrence and the patient remained symptom-free (Figure 7).

## Discussion

OO typically occurs in the long trabecular bones, but is rarely reported in the foot and hallux with incidence rates of less than 2%, including only 17 cases in the great toes and only one pediatric case among them^3^ (Table 1). OO is radiographically classified based on axial distribution of tumor, proposed by Edeiken, classified OO as cortical, cancellous (or medullary), or subperiosteal origin.^4,5^ While cortical origin comprises 75% of all OOs, subperiosteal OOs are the least common type, accounting for less than 5%. However, a study by Kayser et al^5^ proposed a hypothesis that all OOs arise from the subperiosteum and progressively migrate internally.

**Table 1:** Summary of the Available Previous Literature on Osteoid Osteoma in the Great Toe^9^

Number	Author	Year	Sex	Age	Location	Treatment
1	Kahn et al^8^	1983	Female	32	Distal phalanx	Surgical excision
2	Alkalay et al^9^	1987	Male	22	Ossicle	Surgical excision
3	Mohr et al^10^	1990	Male	20	Distal phalanx	Surgical excision
4	Ekmekci et al^11^	2001	Female	29	Subungual	Surgical excision
5	Ozturk et al^12^	2008	Female	9	Distal phalanx	Surgical excision
6	Jowett et al^13^	2010	Female	20	Proximal phalanx, head	Surgical excision
7	Hattori et al^14^	2011	Male	22	Distal phalanx, shaft	Surgical excision
8	Turkmen et al^2^	2013	Male	23	Distal phalanx, shaft	Surgical excision
9	Mohsen et al^15^	2015	Female	32	Subungual	Surgical excision
10	Yamaga et al^16^	2015	Male	16	Distal phalanx	Surgical excision
11	Xarchas et al^17^	2017	Male	22	Distal phalanx	Surgical excision
12	Manae et al^18^	2017	Male	17	Distal phalanx, tip	Surgical excision
13	Torrent et al^19^	2017	Male	16	Proximal phalanx, condyle	Surgical excision with bone autograft
14	Hassini et al^20^	2019	Male	23	Sesamoid	Surgical excision
15	Trave et al^21^	2020	Female	20	Distal phalanx	Surgical excision
16	Basile et al^6^	2020	Female	27	Proximal phalanx, base	Surgical excision
17	Ozdemir et al^22^	2020	Female	46	Distal phalanx, shaft	Radiofrequency ablation

Characteristic clinical manifestations include intermittent pain predominating at night but easily relieved by NSAIDs. The pathophysiology of pain in OOs is due to the presence of nerve endings in the tumor and the high level of prostaglandin and prostacyclin in the nidus. Such high levels of prostaglandin E2 cause inflammatory reactions in the periphery of the tumor and vasodilation, causing bone marrow edema in surrounding tissues, both of which can be radiologically evidenced.^1,6^

The time taken for the correct diagnosis of OO in the great toe is often delayed because of the rarity of the disease and variable radiologic imaging appearances depending on the disease progression. Therefore, the differential diagnosis of OO in the great toe includes subacute osteomyelitis, intraosseous abscess, osteoblastoma, stress fracture, hemangioma, and inclusion epidermoid cyst.^6,7^ Among various diagnostic modalities including plain radiograph, bone scan, CT, and MRI, CT has preoperatively the greatest diagnostic values in demonstrating low-attenuation nidus with localized central calcification and peripheral rim sclerosis because OOs in small bones, unlike typical long bones, may present atypical radiologic presentations, such as medullary origin, absence of osteosclerosis, and multicentric nidi.^1,6^ In this case, MRI was helpful to rule out infection and other OO-mimicking lesions, but the CT scan was integral in obtaining a better understanding of the osseous nature of OO in the unusual location.

The critical point from this case report is associated with clinical suspicion of OO and its differential diagnosis in the uncommon location. Table 1 summarizes the available literature on OOs occurring in the great toe in a chronological order, where its primary treatment modality was surgical excision in all cases.^8,9,10,11,12,13,14,15,16,17,18,19,20,21,22^ In addition to surgical excision of the OO, radiofrequency ablation (RFA) has shown clinically equivalent treatment results with advantages of a minimally invasive procedure, negligible postprocedural complications, low recurrence rates, and a shorter recovery time.^23^ Intraoperative navigation-assisted or CT-assisted RFA is also an excellent noninvasive modality to accurately localize and ablate medullary OOs. However, the use of RFA is often limited when the location of OO is within 1 cm away from critical anatomical structures such as neurovascular structure and skin.^24^ In this case, because the location of the nidus was just below the flexor tendon and near the skin and phalangeal neurovascular structures, the patient underwent en bloc surgical resection to prevent nerve damages and thermal burn from the RFA procedure. In addition, the localization of the nidus was not challenging because of its superficial location to the cortex, and en bloc excision under direct visualization was sufficient for the complete removal in this case.

## Conclusion

In this article, we presented a rare example of juxta-articular and subperiosteal OO occurring in the head of the proximal phalanx of the great toe to the limited literature. The atypical location of OO should be familiarized to include appropriate differential diagnosis and to ensure accurate diagnosis by radiologic evaluations. Surgical excision remains the benchmark for the treatment of OO with its advantages on direct visualization and histologic confirmation for the diagnosis.

## References

[1] Jordan RW Koç T Chapman AW Taylor HP: Osteoid osteoma of the foot and ankle: A systematic review. Foot Ankle Surg 2015;21:228-234. 26564722 10.1016/j.fas.2015.04.005

[2] Turkmen I Alpan B Soylemez S Ozkan FU Unay K Ozkan K: Osteoid osteoma of the great toe mimicking osteomyelitis: A case report and review of the literature. Case Rep Orthop 2013;2013:1-5. 10.1155/2013/234048 PMC3771467 24066250

[3] Basile A Liuni FM Fontanarosa A Zoccali C Baldi J Lanzetti RM: Osteoid osteoma of the proximal phalanx of the hallux: A case report of a challenging diagnosis. Acta Biomed 2020;91:360-364. 32420974 10.23750/abm.v91i2.8493 PMC7569616

[4] Edeiken J DePalma AF Hodes PJ: Osteoid osteoma. (Roentgenographic emphasis). Clin Orthop Relat Res 1966;49:201-208. 5962620

[5] Kayser F Resnick D Haghighi P : Evidence of the subperiosteal origin of osteoid osteomas in tubular bones: Analysis by CT and MR imaging. AJR Am J Roentgenol 1998;170:609-614. 9490939 10.2214/ajr.170.3.9490939

[6] Carneiro BC Da Cruz IAN Ormond Filho AG : Osteoid osteoma: The great mimicker. Insights Imaging 2021;12:32. 33683492 10.1186/s13244-021-00978-8 PMC7940467

[7] Shah J Gandhi D Chauhan A Gupta S: Imaging review of pediatric benign osteocytic tumors and latest updates on management. J Clin Med 2021;10:2823. 34206870 10.3390/jcm10132823 PMC8267885

[8] Kahn MD Tiano FJ Lillie RC: Osteoid osteoma of the great toe. J Foot Surg 1983;22:325-328. 6643942

[9] Alkalay I Grunberg B Daniel M: Osteoid osteoma in an ossicle of the big toe. J Foot Surg 1987;26:246-248. 3611623

[10] Mohr VD Bauer T Schmitt B: Osteoid osteoma at the end of the phalanx of the big toe [in German]. Dtsch Med Wochenschr 1990;115:1470-1474. 2209430 10.1055/s-2008-1065180

[11] Ekmekci P Bostanci S Erdoğan N Akçaboy B Gürgey E: A painless subungual osteoid osteoma. Dermatol Surg 2001;27:764-765. 11493303 10.1046/j.1524-4725.2001.00332.x

[12] Oztürk A Yalçinkaya U Ozkan Y Yalçin N: Subperiosteal osteoid osteoma in the hallux of a 9-year-old female. J Foot Ankle Surg 2008;47:579-582. 19239871 10.1053/j.jfas.2008.07.003

[13] Jowett CR Singh D: Osteoid osteoma of the great toe: A case report. Foot Ankle Surg 2010;16:e12-e15. 20483118 10.1016/j.fas.2009.04.005

[14] Hattori H Takase K Morohashi A: Osteoid osteoma of the great toe. Orthopedics 2011;34:e432-e435. 21815591 10.3928/01477447-20110627-33

[15] Mohsen M Ilaslan H Davis A Sundaram M: Subungual osteoid osteoma of the distal phalanx of the great toe. Orthopedics 2015;38:344-399. 26091208 10.3928/01477447-20150603-01

[16] Yamaga K Minamizaki T Dokai T Kikkawa T Yoshida H: Increasing great toe pain in a patient with soft tissue swelling and nail enlargement. Skeletal Radiol 2015;44:10111065-10671017. 10.1007/s00256-015-2109-5 25666880

[17] Xarchas KC Kyriakopoulos G Manthas S Oikonomou L: Hallux osteoid osteoma: A case report and literature review. Open Orthop J 2017;11:1066-1072. 29151998 10.2174/1874325001711011066 PMC5676009

[18] Al Manae L: Osteoid osteoma of the big toe mimicking epidermal inclusion cyst: A case report and review of the literature. Orthopedics Rheumatol Open Access J 2017;5:1-3.

[19] Torrent J Bailez A Asuncion J: Recurrence of an intra-articular osteoid osteoma of the great toe: A case report and review of the literature. J Surg Case Rep 2017;2017:rjw226. 28064244 10.1093/jscr/rjw226 PMC5219002

[20] Hassini L Khalifa MA Othman Y Grissa Y: Osteoid osteoma of the sesamoid bone: An unusual localization. J Clin Orthop Trauma 2019;10:1065-1067. 31708629 10.1016/j.jcot.2018.10.019 PMC6834943

[21] Trave I Chiarlone F Barabino G Parodi A: Osteoid osteoma of the great toe: Dermatological signs as a disease spy. Int J Dermatol 2020;59:e213-e214. 31970749 10.1111/ijd.14777

[22] Özdemir M Kavak RP Demirler Şimşir B Duman E: Unusually delayed manifestation of a hallux osteoid osteoma: A case report. Int J Surg Case Rep 2020;68:8-11. 32109770 10.1016/j.ijscr.2020.02.032 PMC7044663

[23] Ghanem I: The management of osteoid osteoma: Updates and controversies. Curr Opin Pediatr 2006;18:36-41. 16470160 10.1097/01.mop.0000193277.47119.15

[24] Huang A: Radiofrequency ablation of osteoid osteoma: Difficult-to-Reach places. Semin Musculoskelet Radiol 2016;20:486-495. 28002870 10.1055/s-0036-1594280

